# Age-Related Differences in the Accuracy of Web Query-Based Predictions of Influenza-Like Illness

**DOI:** 10.1371/journal.pone.0127754

**Published:** 2015-05-26

**Authors:** Alexander Domnich, Donatella Panatto, Alessio Signori, Piero Luigi Lai, Roberto Gasparini, Daniela Amicizia

**Affiliations:** 1 Department of Health Sciences, University of Genoa, Genoa, Italy; 2 Inter-University Centre of Research on Influenza and other Transmissible Infections (CIRI-IT), Genoa, Italy; New York City Department of Health and Mental Hygiene, UNITED STATES

## Abstract

**Background:**

Web queries are now widely used for modeling, nowcasting and forecasting influenza-like illness (ILI). However, given that ILI attack rates vary significantly across ages, in terms of both magnitude and timing, little is known about whether the association between ILI morbidity and ILI-related queries is comparable across different age-groups. The present study aimed to investigate features of the association between ILI morbidity and ILI-related query volume from the perspective of age.

**Methods:**

Since Google Flu Trends is unavailable in Italy, Google Trends was used to identify entry terms that correlated highly with official ILI surveillance data. All-age and age-class-specific modeling was performed by means of linear models with generalized least-square estimation. Hold-out validation was used to quantify prediction accuracy. For purposes of comparison, predictions generated by exponential smoothing were computed.

**Results:**

Five search terms showed high correlation coefficients of > .6. In comparison with exponential smoothing, the all-age query-based model correctly predicted the peak time and yielded a higher correlation coefficient with observed ILI morbidity (.978 vs. .929). However, query-based prediction of ILI morbidity was associated with a greater error. Age-class-specific query-based models varied significantly in terms of prediction accuracy. In the 0–4 and 25–44-year age-groups, these did well and outperformed exponential smoothing predictions; in the 15–24 and ≥ 65-year age-classes, however, the query-based models were inaccurate and highly overestimated peak height. In all but one age-class, peak timing predicted by the query-based models coincided with observed timing.

**Conclusions:**

The accuracy of web query-based models in predicting ILI morbidity rates could differ among ages. Greater age-specific detail may be useful in flu query-based studies in order to account for age-specific features of the epidemiology of ILI.

## Introduction

Seasonal influenza is a relatively predictable annual event which causes approximately half a million deaths worldwide every year [1]. It is well established that influenza morbidity is age-related, the greatest attack rates usually being observed in the pediatric population [2–4]. Indeed, we previously showed [5] that in Italy, in two consecutive post-pandemic influenza seasons, the highest influenza-like illness (ILI) morbidity rate was documented among children under 14 years of age. Moreover, the timing of influenza peaks may vary across age-groups. Some studies have highlighted a crucial role of young children, especially preschool children, in spreading influenza in households [6] and have shown that these subjects display the earliest peak during influenza epidemics [7]. On the other hand, Glass et al. [8] pointed out the importance of high-school students in the local spread of the virus, while Schanzer et al. [9] doubted the hypothesis that younger school-age children drive epidemic waves. Specifically, these latter authors demonstrated that, during influenza seasons in which H3N2 strains predominated, young adults aged 20–29 years led teenagers aged 10–19 years by about 4 days, while during the last pandemic this latter group led both 4–9-year-olds and young adults [9]. Understanding such age patterns is of interest, as it can help to prioritize the use of limited supplies of vaccines and antiviral drugs [10].

The early detection of outbreaks of disease, including influenza, is crucial to minimizing their spread and reducing the disease-associated burden [11]. The spread of influenza at the community level can be tracked by monitoring laboratory-confirmed cases, cases diagnosed by general practitioners (GPs), attendances at emergency departments, hospital admissions and excess deaths [12]. However, in the last few years an increasing number of literature reports have emphasized the usefulness of collecting and mining web data on notifiable disease surveillance, including, for example, tuberculosis [13], dengue [14], HIV and sexually transmitted infections [13, 15], tick-borne diseases [16] and influenza and ILI [17–29]. This novel epidemiological approach has been conceptualized as a way of studying “…distribution and determinants of information in an electronic medium… or in a population, with the ultimate aim to inform public health and public policy” [30], and has been widely applied to surveillance and the analysis of trends. One of the first studies carried out in Canada [19] found a high correlation between cases of ILI reported by sentinel physicians and the number of clicks on a keyword-triggered link in one of the Google services. An open tool for real-time influenza surveillance, Google Flu Trends (GFT), was subsequently launched in November 2008 [17, 18] and somewhat popularized the analysis of influenza-related online activity to track ILI at the population level [30]. Apart from the most widely explored GFT, the use of other online tools and data sources for web-based ILI surveillance has been proposed, including search engines other than Google [20, 25] and web sites [23], Twitter [26, 27, 29], Wikipedia [28], Google Correlate [31] and Google trends (GT) [22, 24]; this last is methodologically similar to GFT but requires the use of *ad hoc* search terms. Indeed, as of February 2015, GFT is available in only 29 countries. While most of the above-mentioned studies confirmed the utility of flu query-based surveillance by documenting a high correlation and prediction accuracy, others pointed out some major limitations [18, 31–34]. In particular, two recent papers [33, 34] reported that GFT significantly overestimated influenza activity in the United States. However, prediction accuracy may be improved by combining data from both GFT and the Centers for Disease Control and Prevention (CDC) rather than using GFT data alone [34]. Another concern regards the earlier peak of flu incidence estimated by search volume in comparison with traditional surveillance, which is very probably due to the influence of the media [32]. Lack of transparency, the impossibility of accounting for trends of single queries (as a result of combining many queries into a single variable), opinion-based exclusion of queries from the updated GFT and the static nature of the model, which ignores changes in search behavior, have also been cited among the weaknesses of GFT [31].

Large population-based surveys [35] have revealed that using the Internet to search for health-related topics varies across age-classes; surfing the web for symptoms, for example, is highest among middle-age adults. There is also some age-related difference in the primary purpose of online searching, in terms of looking for information related to one’s own health or on behalf of somebody else. Indeed, online health information seekers aged over 65 years are more likely to search on their own behalf [35].

Despite the above-described age-related patterns in online information seeking, little is known about whether the association between ILI-related queries and ILI morbidity is homogeneous across age-classes or not. Indeed, in one study [36], correlation coefficients between GFT and both the number of positive influenza tests and emergency department ILI visits were found to be substantially higher among adults than among pediatric patients. This paper therefore aimed to investigate how well ILI-related queries submitted to the most popular search engine may predict age-class-specific ILI morbidity rates.

## Methods

### ILI morbidity data

Weekly ILI morbidity was recorded from data collected by the Inter-University Centre for Research on Influenza and other Transmissible Infections (CIRI-IT), Genoa (Italy) [37]. The CIRI-IT is one of the two reference centers of the Italian sentinel surveillance of influenza (Influnet), which covers over two percent of the Italian population. Sentinel GPs and independent pediatricians send reports (including zero reports) of ILI cases diagnosed among their patients on a weekly basis [5, 38]. ILI is defined as the abrupt onset of fever of 38°C or more, at least one respiratory symptom (non-productive cough, sore throat, rhinitis) and at least one systemic symptom (headache, myalgia and severe malaise) [5]. The CIRI-IT coordinates the activities of sentinel physicians from nine of 20 Italian regions (Liguria, Lombardy, Friuli Venezia Giulia, Tuscany, Umbria, Abruzzo, Apulia, Calabria and Sicily). In order to better analyze ILI dynamics among adults, the CIRI-IT recorded age-specific ILI morbidity data from the 2011/2012 (42^nd^ week of 2011) influenza season in a more detailed way, i.e. by subdividing the population into six age-classes (0–4, 5–14, 15–24, 25–44, 45–64 and ≥ 65 years) instead of four (0–4, 5–14, 15–64 and ≥ 65 years). Overall and age-class-specific ILI morbidity data, expressed as the number of cases per 100,000 inhabitants, were recorded from the 42^nd^ week of 2011 to the 8^th^ week of 2015 (175 weeks).

### Query volume and selection of search terms

To date (February 2015), GFT is not available in Italy. Query volume (QV) was therefore assessed by means of GT. On GT, weekly search volume data from October 2011 to February 2015 were extracted (on February 27, 2015); these were regarded as ILI morbidity data. GT analyzes selected web queries (provided that their search volume is sufficient) and displays the results on a normalized scale ranging from 0 to 100 [24]. Search terms were identified through two steps. First, GT was explored by searching for ILI-related terms identified on the basis of common knowledge of the disease, Google autocomplete service and previous research [22, 24]. QVs of selected terms, downloaded one by one, were then correlated with CIRI-IT data. Second, since GT only enables up to five entry terms to be inserted at a time, these five were selected from among those showing the highest correlation coefficients at the previous step (in order to account for the difference in magnitude of their search volume) [Data Source: Google Trends (www.google.com/trends)].

### Statistical analysis and modeling

Pearson’s correlation coefficient with 95% confidence intervals (CIs) was used to evaluate the correlation between the relative QV and ILI morbidity. The whole dataset was split into a training set—comprising three years (156 weeks: from 42^nd^ week of 2011 to 41^st^ week of 2014)—and a hold-out validation set (the remaining 19 weeks). Since the dependent variable “ILI morbidity” was highly positively skewed, the square-root transformation was applied in order to obtain a more symmetric distributions. Modeling of QV data may be challenging, owing to the serial correlation of residuals [34]. To take autocorrelation into account, models using asymptotically efficient generalized least-squares (GLS) estimation with residuals following the (*p*, *q*) autoregressive-moving-average (ARMA) process (where *p* and *q* determine the order of the process) were constructed. Model selection was performed in two main steps. Firstly, the best subset of independent variables—i.e. the one that minimized the corrected Akaike Information Criterion (AICc)—was identified among all possible regressions estimated by means of an ordinary least squares (OLS) approach. If two or more competing models showed approximately equal AICc (Δ AICc < 2), the more/most parsimonious one was preferred. Secondly, to find an optimal (*p*, *q*) order for the ARMA process, residual autocorrelations of the preliminary OLS model were examined by plotting autocorrelation and partial autocorrelation functions [39]. In addition, GLS models with alternative ARMA orders and estimation methods (maximum-likelihood or restricted maximum-likelihood) were compared by means of AIC. Model selection, estimation and hold-out validation procedures were performed for each age-class separately. Hold-out predictions made by Holt-Winters exponential smoothing were assessed for comparative purposes. The prediction accuracy of age-class-specific models in hold-out sets was quantified by mean absolute error (MAE), root-mean-square error (RMSE) and Pearson’s *r*. The difference in the prediction accuracy of GLS and exponential smoothing models was formally tested by means of the Diebold-Mariano test. Statistical significance was set to two-sided *p* < .05 All data analyses and modeling were performed by means of the R stats package, version 3.1.2 [40]. Raw data used for the analysis are reported in the Dataset A in S1 File.

## Results

The QV of 18 individual search terms was retrieved (Table 1). Of these, five entry terms, namely “Influenza”, “Fever”, “Cough”, “Tachipirina” (a popular brand name of Paracetamol) and “Paracetamol” (henceforth referred to as *Influenza*, *Fever*, etc) showed high correlation coefficients of > .6 with CIRI-IT morbidity data. These five entry terms were therefore reinserted into GT all together and their relative QV was downloaded.

**Table 1:** Pearson’s correlation coefficient between ILI morbidity and query volume of selected entry terms.

Search term		*r*	95% CI
English spelling	Italian spelling		
*Flu*/*Influenza*	*Influenza*	.882	.844–.911
*Fever*	*Febbre*	.712	.630–.778
*Cough*	*Tosse*	.643	.546–.722
–	*Tachipirina* ^a^	.657	.564–.734
*Paracetamol*	*Paracetamolo*	.607	.504–.693
*Aspirin*	*Aspirina*	.598	.493–.685
*Common cold*	*Raffreddore*	.460	.334–.569
*Stuffy nose*	*Naso chiuso*	.449	.322–.560
*Antibiotic*	*Antibiotico*	.422	.291–.536
*Sore throat*	*Mal di gola*	.370	.235–.492
*Chills*	*Brividi*	.250	.105–.384
*H3N2*	*H3N2*	.188	.041–.327
*Rhinitis*	*Rinite*	-.050	-.197–.099
*H1N1*	*H1N1*	n/a^b^	–
*Oseltamivir*	*Oseltamivir*	n/a^b^	–
*Tamiflu*	*Tamiflu*	n/a^b^	–
*Bird flu*	*Influenza aviaria*	n/a^b^	–
*Swine flu*	*Influenza suina*	n/a^b^	–

^a^: Popular brand name of Paracetamol;

^b^: Only monthly data were available

**Fig 1:**
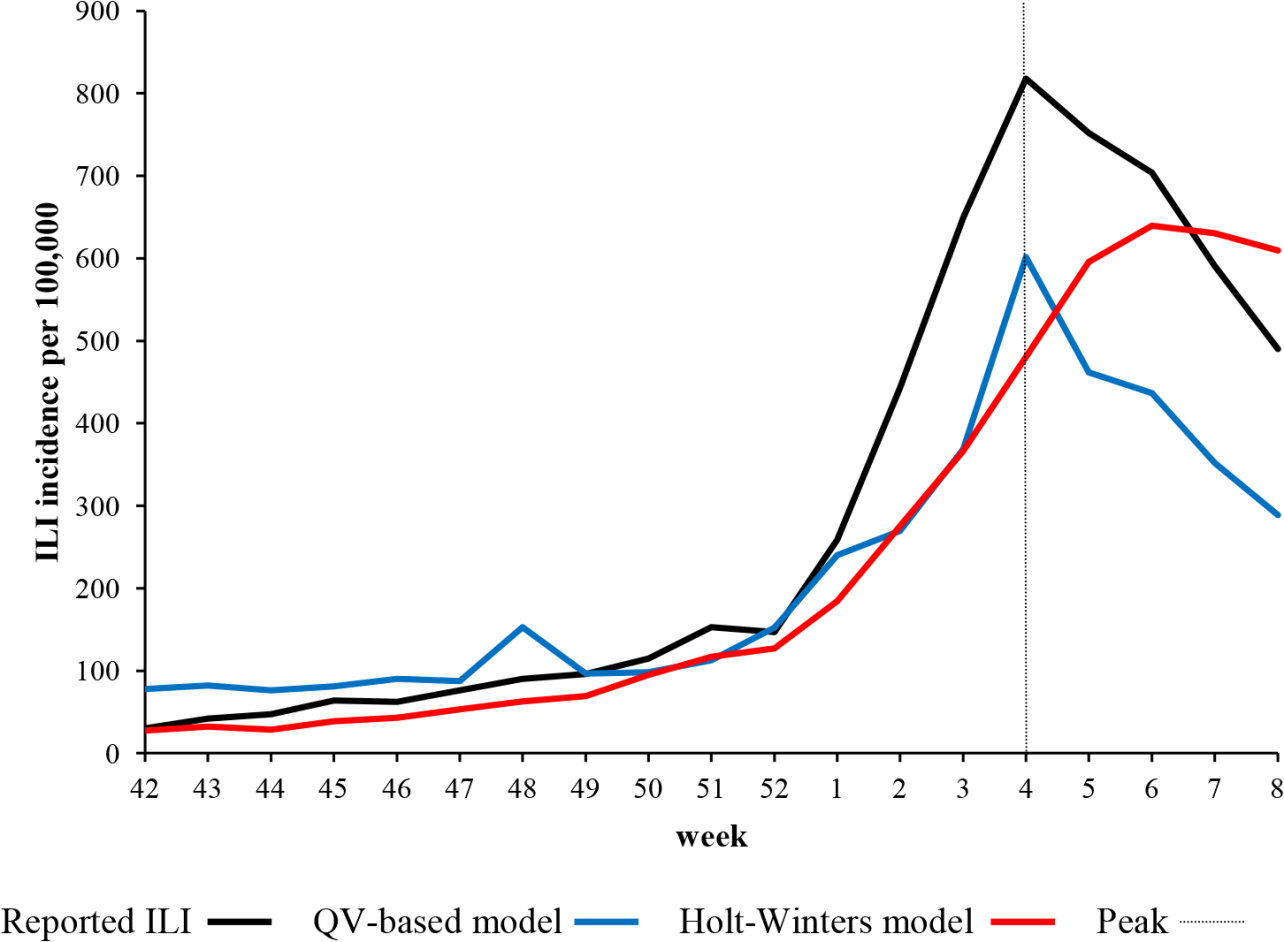
Time-series of reported versus predicted all-age ILI morbidity (hold-out validation). Reported all-age ILI morbidity compared with hold-out predictions (from 42^nd^ week of 2014 to 8^th^ week of 2015) generated by the query-based model and exponential smoothing.

Among several QV-based candidate models for predicting overall ILI morbidity, we chose a model in which the independent variables were *Influenza*, *Fever* and *Tachipirina* and residuals followed the ARMA(1,0) process. The maximum likelihood estimation of the error-autoregressive parameter was sizable. During the validation stage, prediction based on the selected QV model was associated with greater errors (Δ RMSE 27%) in comparison with Holt-Winters exponential smoothing (Table 2). The between-model difference in prediction accuracy was, however, not statistically significant. By contrast, the QV-based model was able to correctly predict the peak time (4^th^ week), although the height of the peak was significantly underestimated (Table 3 and Fig 1). QV-based prediction yielded a higher correlation coefficient with the official ILI data [.978 (95% CI: .942–.992)] than the Holt-Winters model [.929 (95% CI: .821–.973)]. In Fig 1, a significant spike (overestimation of about 70%) can be seen at the 48^th^ week in the QV-based prediction time-series.

**Table 2:** Hold-out validation of models to predict all-age ILI morbidity: comparison in terms of MAE and RMSE.

Parameter	Model	Estimate
**MAE**	QV-based	2.87
	Holt-Winters	2.17
**RMSE**	QV-based	3.55
	Holt-Winters	2.79
**Diebold-Mariano test, *p***		.15

**Table 3:** Hold-out validation of models to predict all-age ILI morbidity: comparison in terms of peak timing and peak magnitude.

Parameter	Model	Estimate
**Δ times of peak, weeks**	QV-based	0
	Holt-Winters	+2
**Δ peak height, %**	QV-based	-29.3
	Holt-Winters	-21.8

**Fig 2:**
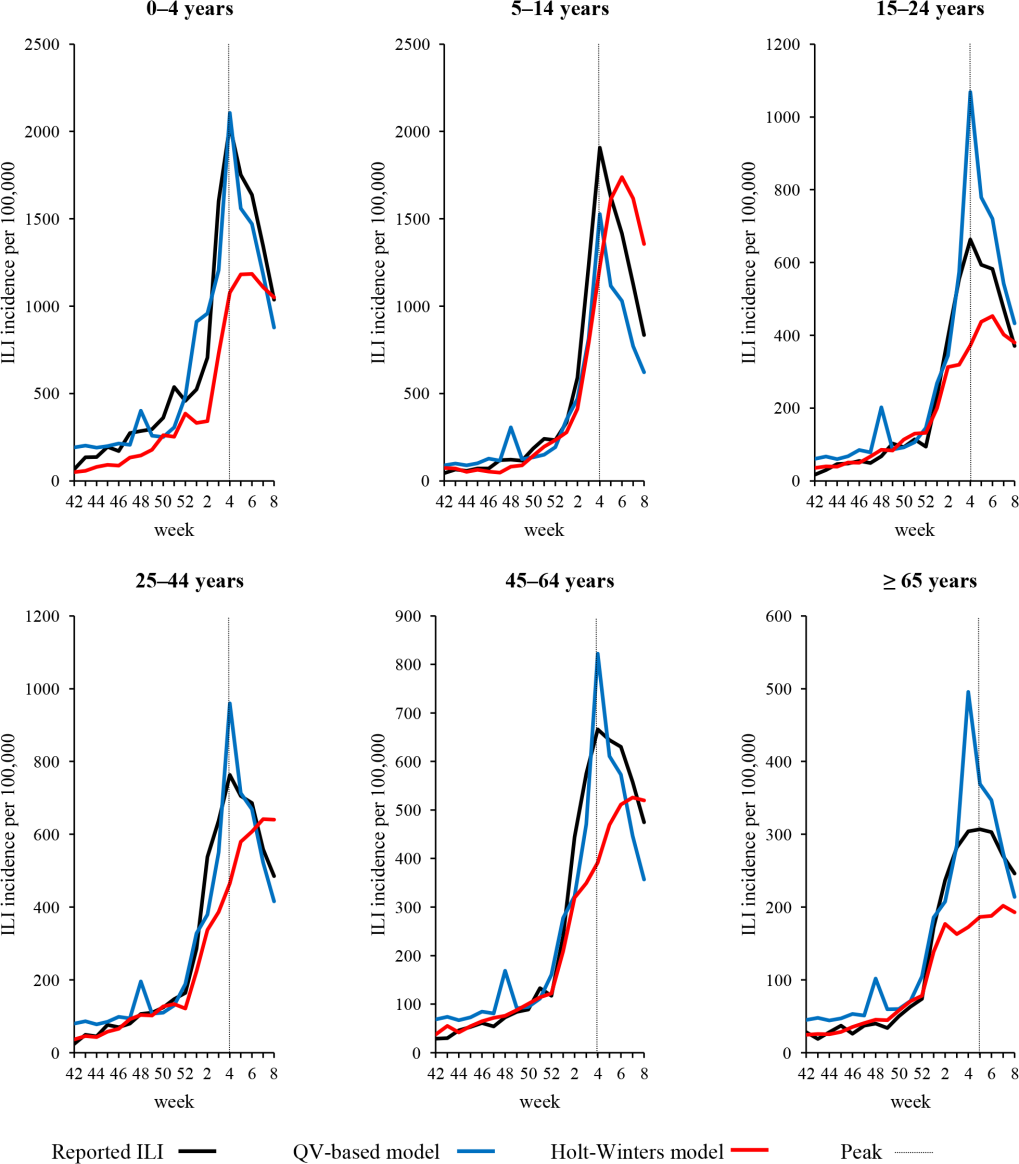
Time-series of reported versus predicted age-class-specific ILI morbidity (hold-out validation). Reported age-class-specific ILI morbidity compared with hold-out predictions (from 42^nd^ week of 2014 to 8^th^ week of 2015) generated by the age-class-specific query-based models and exponential smoothing.

The set of explanatory variables of QV-based models selected for the out-of-sample validation of age-class-specific ILI morbidity was the same as that used for the all-age data (i.e. *Influenza*, *Fever* and *Tachipirina*). Full model specifications and parameter estimates are reported in Table A in S1 File. As shown in Fig 2 and Table 4, the prediction accuracy of six QV-based models varied substantially among the age-classes. Thus, in the 0–4 and 25–44-year age-groups, prediction errors produced by the QV-based models were lower than those produced by Holt-Winters models. A particularly accurate prediction was observed in the youngest age-class, with about a 75% reduction in RMSE in comparison with exponential smoothing errors; the Diebold-Mariano test confirmed a significantly different level of prediction accuracy (*p* = .036). In the 0–4-year age-class, the QV-based model outperformed the competing model in predicting the peak magnitude and only slight (< 5%) incidence overestimation was observed (Table 5). Conversely, it can be seen (Fig 2) that QV-based predictions of ILI morbidity in the age-classes of adolescents and young adults (15–24 years) and the elderly were poorer, especially with regard to the peak incidence (over 60% overestimation), although mean prediction errors were roughly comparable (*p* > .5; Diebold-Mariano test) to those generated by the Holt-Winters models. Similarly, in other age-groups (i.e. 5–14, 15–24, and 45–64 years), the prediction accuracy of QV-based models did not differ significantly from that of exponential smoothing, although the former tended to overestimate and the latter to underestimate ILI peak activity. Despite an observed tendency of QV-based models to overestimate ILI incidence (Fig 2), their predictions of peak time matched CIRI-based estimates in all but one (over-64-year-olds) age-class. By contrast, peak time predictions of exponential smoothing were delayed by 1–3 weeks (Table 5). All predicted age-class-specific incidence rates were very highly correlated (> .9) with CIRI-IT-based ILI estimates, regardless of the model type (Table B in S1 File).

**Table 4:** Hold-out validation of models to predict age-class-specific ILI morbidity: comparison in terms of MAE and RMSE.

Parameter	Model	Age-class, years					
		0–4	5–14	15–24	25–44	45–64	≥ 65
**MAE**	QV-based	2.90	3.21	2.15	1.60	1.91	1.49
	Holt-Winters	5.08	2.89	1.80	1.79	1.57	1.53
**RMSE**	QV-based	3.46	3.86	2.79	2.02	2.19	1.89
	Holt-Winters	6.05	3.97	2.47	2.55	2.34	2.07
**Diebold-Mariano test, *p***		.036	.88	.54	.33	.79	.67

**Table 5:** Hold-out validation of models to predict age-class-specific ILI morbidity: comparison in terms of peak timing and peak magnitude.

Parameter	Model	Age-class, years					
		0–4	5–14	15–24	25–44	45–64	≥ 65
**Δ times of peak, weeks**	QV-based	0	0	0	0	0	-1
	Holt-Winters	+2	+2	+2	+3	+3	+2
**Δ peak height, %**	QV-based	3.6	-19.9	61.1	25.8	23.4	61.5
	Holt-Winters	-41.7	-8.8	-31.7	-15.9	-21.0	-34.2

## Discussion

Influenza-related morbidity and its impact are highly age-specific, suggesting the need for more detailed age data in surveillance systems [3]. The present paper explored patterns in the association between ILI-related online queries and age-specific ILI morbidity. We found a perceptible difference in the accuracy of predicting age-class-specific ILI morbidity from GT QV. Specifically, QV-based models performed well in the age-classes of children aged 0–4 years and adults aged 25–44 years, while ILI predictions in 15–24-year-olds and the elderly were subject to high errors. Given that ILI-related QV cannot be differentiated by age, an exact interpretation of these observations is difficult. They could be explained by the age-based digital divide and the age-distinct purposes of web searching for health information [35]. In the former case, it is well-known that elderly people are less likely to use the Internet than younger age-groups [35, 41]. Although most teenagers and young adults are active web users [42], they may make less use of online searching for influenza-related content. Indeed, the large National Health Interview Survey [41] documented a lower use of the Internet for health purposes among 18–24-year-olds (46.5%) than among other, non-elderly, adult groups (25–34 years: 55.1%; 35–44 years: 52.2%; 45–64 years: 47.6%). Moreover, Paolotti et al. [43] and Debin et al. [44] have established that users of a web-based real-time participatory influenza surveillance system are not representative of the age structure of the Italian and French populations, respectively. Specifically, under-20-year-olds and elderly users of the Italian platform Influweb were highly underrepresented in the Internet-based cohort (7.4% vs. 19% and 10.6% vs. 24.8% of the general population, respectively) [43]. We believe that the above considerations could contribute to the relatively poor performance in predicting ILI in the 15–24 and ≥ 65-year age-classes. By contrast, in ≤ 4 and 25–44-year age-groups, models based on GT did well and even outperformed the widely used Holt-Winters method. Middle-aged adults are not only the most active internet users but also most frequently search the web to answer their health-related queries [35, 41]. In the case of young children, it is obvious that an ILI-related web search may be conducted by a “worried” parent. Indeed, research has shown that the Internet is one of the main sources of child health information, as most parents surf the web for medical information, especially when their child is sick [45–47]. Parents of children ≤ 3 years of age tend to search the web more than parents of older ones [48]. Interestingly, parents tend to Google their child’s condition rather than symptoms [49].

In sum, the natural attack rates of influenza may display a marked difference among age-classes [5, 50]. An accurate prediction of age-specific ILI morbidity rates is of importance to public health, as it may, for instance, help to rationalize vaccine supplies for the age-groups recommended for immunization; forecasting age-specific ILI attack rates should therefore be undertaken through age-specific approaches. The anonymous statistics on queries submitted to popular search engines do not currently allow us to trace the demographics of those who search for a given topic. In this regard, web-based participatory systems of influenza surveillance, such as Influenzanet [51] or its regional partners (e.g. Italian Influweb [52]), provide an advantage as they are able to distinguish their estimates by age-class and adjust ILI attack rates for age in order to correct for underrepresented age-classes; the prospective nature of the cohorts also favors these portals. However, the overall statistics of QV from the common search engines, such as Google, have a much wider coverage at the population level (for comparison: as of March 12, 2015, Influweb had 3,740 volunteers [52]). Establishing the purpose of ILI-related online searching among subjects of different ages will further contribute to our understanding of the relationship between online queries and ILI incidence. For example, before analyzing GT, Cho et al. [22] asked patients what entry terms they would use if they were searching the web for influenza. A similar survey involving people of different ages would be helpful, as elderly Internet users less frequently make use of search engines [53] or look for a specific disease or medical problem [35] than younger individuals.

Some important remarks and comparisons with previous research regarding the accuracy of our estimates of all-age ILI morbidity should be made. GFT has been shown to have significantly overestimated CDC data in the consecutive influenza seasons 2011/2012 [34], 2012/2013 [18, 33, 34] and 2013/2014 [54]. Apparently, this is in contrast with our findings, in which peak height was significantly underestimated by about 30%. It should be borne in mind, however, that in the 2014/2015 influenza season in Italy overall ILI morbidity was significantly higher and peaked earlier (4^th^ week of 2015) than in the previous three seasons, and was at the level recorded in the 2010/2011 post-pandemic period. GFT underestimation of ILI activity was also seen earlier; the original GFT model underestimated ILI at the beginning of the last pandemic [18, 33]. Although our QV-based model did not produce accurate predictions of overall ILI incidence, its estimate of the timing of peak activity coincided with the official surveillance data and yielded a very high correlation coefficient with CIRI-IT-based data; this is consistent with a great body of previous research [55, 56].

More generally, as far as we know this is the first study to investigate the application and utility of GT QV for near-real-time ILI surveillance in Italy. Overall, our correlation analysis revealed that ILI-related QV, especially *Influenza*, is highly correlated with official surveillance data; this is in line with previous research conducted in different settings [21–29]. However, some noteworthy differences arose between our results and those of studies [22, 24] from regions where GFT is unavailable. In a study conducted in South Korea [22], the correlation coefficient between the GT QV of *Flu* and both ILI morbidity and virological surveillance was much lower, or even negative, in the 2008/09 influenza season. A similarly low strength of correlation between the search term *Flu* and ILI attack rates was reported in Southern China [24]. On the other hand, the studies by Cho et al. [22] and Kang et al. [24] were able to identify other entry terms, such as *Bird flu* or *Tamiflu*, which proved to be highly correlated with both virological and syndromic surveillance data. Again, such inconsistence may be due to the different study periods, since both Asian studies included the pandemic period, which could affect search behavior. Indeed, in our study, the QV of *H1N1* was insufficient on a weekly basis, while in both Asian studies this entry term was investigated. Another possible explanation may regard cross-cultural differences and variations in the Internet penetration rate.

Apart from the well-known shortcomings of web-based surveillance methods, which are described elsewhere [30, 57], the present study may have other limitations; our results should therefore be interpreted cautiously. First, the catchment area of the CIRI-IT encompasses nine (of 20) regions, roughly corresponding to 50% of the Italian population. However, our syndromic surveillance data are fairly representative of the whole population, since they come from Northern, Central, Southern and Insular Italy. Moreover, the correlation between CIRI-IT data and the nationwide Influnet data (which is available for only 28 weeks) [58] during the last three influenza seasons was 0.996–0.999 (results not shown). Second, only a few entry terms were used in the analysis. These, however, may be seen as an information concepts [57] and not as simple keywords, since, according to the Google trends instructions [59], typing the word *Influenza* without quotation marks, for example, will also include related searches, such as *I’ve got influenza*, *Influenza symptoms* or *Influenza remedies*. Third, our models did not take into account media activity, which may lead to an increase in ILI-related searches [17]. For instance, GFT may correlate positively with the number of both television broadcasts and newspaper articles [60]. Our QV-based predictions showed a substantial overestimation of ILI activity in the 48^th^ week (last week of November) of 2014. In Italy, a series of alleged post-flu shot deaths were reported in the elderly in this period (no causal association was later established) and on November 27 two batches of a vaccine were suspended for precautionary purposes [61]. On the other hand, this “false alarm” was not able to shift the peak time, and QV-based predictions peaked in the same week as in the data from syndromic surveillance. Fourth, as has been suggested by Santillana et al. [31], the web-searching behavior of Google users may change over time; prediction models should therefore be dynamic enough to capture these changes. In this regard, our models are rather static and may not be generalizable to long-term forecasts or to other geographical settings. Fifth, since GT displays only the relative QV, its values may change over time and/or on using different keyword comparators.

In conclusion, it is unlikely that web-based techniques of ILI surveillance will substitute traditional surveillance methods in the near future and the former should be seen as a low-cost near-real-time complementary source to the latter. In Italy, digital ILI detection may have a certain value: among users of the Influweb portal, only 55% and 4% phoned and visited, respectively, their GP during an ILI episode [43]. A better understanding of how people of different ages exploit common search engines to find ILI-related information, together with greater age-specific detail, may be useful in future web query-based studies and field implementation of digital disease surveillance techniques, in order to take into account age-specific features of the epidemiology of ILI.

## Supporting Information

S1 File This file contains results of linear models using generalized least squares estimation to predict all-age and age-class-specific ILI morbidity (Table A), Pearson’s correlation coefficients between predicted and reported ILI morbidity by age-class (Table B), raw data used for the analysis (Dataset A) and an example of R code (Box A). (PDF)
