# Supplementary material for: Age-Related Differences in the Accuracy of Web Query-Based Predictions of Influenza-Like Illness
Source: PLoS One. 2015 May 26;10(5):e0127754. doi: 10.1371/journal.pone.0127754 (PMC4444192; doi:10.1371/journal.pone.0127754)
Supplement: S1 File — (PDF) [file pone.0127754.s001.pdf]

Supporting Information File S1.

Table A. Results of the linear models using generalized least squares estimation to predict all-age and age-class-specific ILI morbidity.

Model/age-class, years	Parameter	Estimate	SE	$\|t\|$	p
**All-age**[a]	b_0	3.778	2.446	1.54	.12
	b_1	0.198	0.036	5.51	<.001
	b_2	0.037	0.033	1.14	.26
	b_3	-0.051	0.057	0.90	.37
	φ	0.954	–	–	–
**0–4**[a]	b_0	2.659	4.770	0.56	.58
	b_1	0.380	0.085	4.45	<.001
	b_2	-0.030	0.078	0.39	.70
	b_3	0.197	0.136	1.45	.15
	φ	0.935	–	–	–
**5–14**[a]	b_0	3.038	3.739	0.81	.42
	b_1	0.441	0.085	5.22	<.001
	b_2	0.082	0.081	1.01	.31
	b_3	-0.333	0.139	2.38	.018
	φ	0.881	–	–	–
**15–24**[b]	b_0	1.661	2.444	0.68	.50
	b_1	0.354	0.055	6.38	<.001
	b_2	0.067	0.072	0.94	.35
	b_3	-0.225	0.124	1.82	.070
	φ	0.841	–	–	–
	ψ	-0.397	–	–	–
**25–44**[b]	b_0	2.089	2.414	0.87	.39
	b_1	0.282	0.048	5.85	<.001
	b_2	0.050	0.051	0.99	.33
	b_3	-0.083	0.087	0.95	.35
	φ	0.935	–	–	–
	ψ	-0.265	–	–	–
**45–64**[c]	b_0	2.007	2.112	0.95	.34
	b_1	0.262	0.049	5.41	<.001
	b_2	0.049	0.051	0.95	.34
	b_3	-0.086	0.088	0.98	.33
	φ_1	0.671	–	–	–
	φ_2	0.192	–	–	–
**≥ 65**[d]	b_0	1.713	1.658	1.03	.30
	b_1	0.196	0.039	5.02	<.001
	b_2	0.017	0.046	0.37	.71
	b_3	-0.007	0.079	0.09	.93
	φ_1	0.478	–	–	–
	φ_2	0.309	–	–	–

[a]: $\sqrt{\text{ILI}_t} = b_0 + b_1 \cdot Influenza_t + b_2 \cdot Fever_t + b_3 \cdot Tachipirina_t + \varphi\varepsilon_{t-1} + \nu_t$ (maximum-likelihood estimation);

[b]: $\sqrt{\text{ILI}_t} = b_0 + b_1 \cdot Influenza_t + b_2 \cdot Fever_t + b_3 \cdot Tachipirina_t + \varphi\varepsilon_{t-1} + \nu_t + \psi\nu_{t-1}$ (restricted maximum-likelihood estimation);

[c]: $\sqrt{\text{ILI}_t} = b_0 + b_1 \cdot Influenza_t + b_2 \cdot Fever_t + b_3 \cdot Tachipirina_t + \varphi_1\varepsilon_{t-1} + \varphi_2\varepsilon_{t-2} + \nu_t$ (maximum-likelihood estimation);

[d]: $\sqrt{\text{ILI}_t} = b_0 + b_1 \cdot Influenza_t + b_2 \cdot Fever_t + b_3 \cdot Tachipirina_t + \varphi_1\varepsilon_{t-1} + \varphi_2\varepsilon_{t-2} + \nu_t$ (restricted maximum-likelihood estimation).

Table B. Pearson's correlation coefficients between predicted and reported ILI morbidity by age-class and model type (all p < .001).

Age-class, years	QV-based model		Holt-Winters model	
	r	95% CI	r	95% CI
0–4	.960	.897–.985	.939	.845–.977
5–14	.988	.968–.995	.906	.768–.964
15–24	.962	.902–.986	.958	.892–.984
25–44	.963	.904–.986	.917	.793–.968
45–64	.956	.887–.983	.953	.879–.982
≥ 65	.942	.853–.978	.975	.935–.991

Dataset A. Raw data used for the analysis.

Year	Week	M099y	M04y	M514y	M1524y	M2544y	M4564y	M6599y	GTinf	GTfev	GTcou	GTtac	GTpar
2011	42	51	174	60	36	44	48	25	19	34	26	11	4
2011	43	54	247	56	62	50	34	18	18	33	29	12	5
2011	44	50	215	53	59	34	38	24	19	36	30	11	4
2011	45	68	250	64	41	72	55	39	18	35	28	11	4
2011	46	72	271	76	50	74	59	29	21	36	30	11	4
2011	47	85	285	79	73	93	72	41	23	36	24	12	7
2011	48	96	342	104	52	102	82	48	22	40	27	14	5
2011	49	98	363	128	91	99	69	45	21	39	30	14	3
2011	50	129	390	154	101	154	107	50	19	41	25	12	4
2011	51	151	547	198	120	158	115	54	23	40	22	14	6
2011	52	214	766	236	174	211	181	109	36	49	32	21	7
2012	1	219	464	233	175	254	216	124	35	56	34	17	7
2012	2	297	611	348	245	319	281	189	32	42	31	16	6
2012	3	466	1423	996	314	393	316	186	35	49	31	18	6
2012	4	643	2088	1532	422	554	410	191	46	53	33	19	6
2012	5	709	2132	1512	469	618	508	271	42	52	33	17	6
2012	6	682	1980	1385	435	634	511	290	36	49	33	17	5
2012	7	575	1565	1052	427	521	476	253	34	49	30	16	5
2012	8	485	1196	813	370	469	383	259	31	51	30	19	6
2012	9	343	821	504	294	348	296	191	29	48	30	15	4
2012	10	246	615	410	240	222	199	133	24	44	28	14	7
2012	11	179	463	311	156	171	137	93	22	40	25	14	4
2012	12	121	407	208	73	109	88	57	17	40	25	12	4
2012	13	93	269	136	89	89	72	48	13	38	24	15	4
2012	14	70	207	96	63	69	52	35	14	34	22	11	3
2012	15	64	168	73	67	71	57	29	15	40	21	11	3
2012	16	58	166	61	46	65	45	34	15	38	22	12	4
2012	17	43	95	52	33	45	36	31	12	37	20	11	4
2012	18	43	201	79	30	39	25	13	13	36	21	12	4
2012	19	14	80	19	0	11	14	9	12	38	19	10	4
2012	20	29	78	0	14	23	43	27	11	41	19	10	4
2012	21	17	52	41	17	6	21	7	10	40	19	10	4
2012	22	23	31	52	0	34	7	25	11	38	19	12	4
2012	23	28	71	60	62	29	9	11	10	40	15	9	4
2012	24	12	34	0	0	19	0	21	10	37	17	10	4
2012	25	14	0	0	75	34	0	0	9	39	15	10	3
2012	26	3	0	0	0	0	10	0	8	41	13	11	4
2012	27	11	0	0	0	0	0	45	6	43	12	11	2
2012	28	7	0	0	0	14	0	15	6	42	12	11	3
2012	29	19	0	39	0	15	15	32	8	38	12	11	4
2012	30	17	155	28	0	11	0	12	8	37	13	9	3
2012	31	8	0	0	45	0	0	17	6	42	13	9	3
2012	32	4	0	0	49	0	0	0	8	37	12	10	4
2012	33	9	0	0	0	0	35	0	7	45	15	13	4
2012	34	0	0	0	0	0	0	0	8	40	15	11	4
2012	35	0	0	0	0	0	0	0	9	39	15	12	3

2012	36	9	0	0	0	21	18	0	13	35	15	10	4
2012	37	17	0	67	0	0	25	14	13	34	17	9	4
2012	38	39	0	0	112	21	59	25	14	37	21	13	4
2012	39	31	135	66	33	36	11	14	17	40	22	14	4
2012	40	38	84	24	73	54	40	0	17	39	24	12	5
2012	41	61	181	172	70	33	57	25	20	39	27	12	4
2012	42	34	116	23	20	38	30	21	19	40	26	14	5
2012	43	48	162	38	41	53	42	26	20	39	27	13	4
2012	44	43	160	28	29	50	35	25	19	38	28	13	5
2012	45	56	185	49	54	62	42	33	21	39	30	14	4
2012	46	62	215	54	49	69	46	36	19	39	29	14	5
2012	47	73	254	60	52	95	56	33	21	42	29	14	4
2012	48	85	251	100	66	107	60	37	19	43	31	15	4
2012	49	93	342	107	85	100	70	37	18	39	28	15	4
2012	50	125	442	182	110	127	86	55	21	41	30	16	4
2012	51	155	645	263	114	127	101	69	23	46	34	17	5
2012	52	156	610	287	112	117	103	76	37	55	47	25	7
2013	1	227	551	279	158	250	199	139	40	61	44	24	7
2013	2	328	642	433	284	355	329	165	49	54	41	21	5
2013	3	438	1162	967	279	399	354	158	59	56	40	21	5
2013	4	577	1437	1524	346	501	418	176	49	60	40	20	5
2013	5	698	1736	1871	418	629	481	208	52	59	42	24	6
2013	6	725	1717	1790	451	634	574	227	55	62	44	25	5
2013	7	721	1587	1633	466	685	635	230	48	62	39	27	6
2013	8	697	1608	1377	462	679	624	252	51	66	39	26	6
2013	9	565	1262	1126	398	559	495	202	43	59	35	23	5
2013	10	423	917	743	341	427	390	168	37	58	35	22	5
2013	11	295	570	505	241	305	272	132	26	50	32	19	4
2013	12	226	511	364	187	237	201	86	22	48	30	15	4
2013	13	159	378	270	128	169	141	52	18	45	27	17	4
2013	14	106	172	157	121	120	100	43	18	46	27	15	5
2013	15	81	204	122	74	77	68	43	16	43	27	19	5
2013	16	56	129	81	37	66	43	36	15	45	28	16	5
2013	17	49	157	85	41	39	39	23	12	42	28	14	4
2013	18	26	60	36	38	22	22	19	12	41	27	15	4
2013	19	30	0	57	65	20	32	23	11	40	31	12	4
2013	20	33	0	42	77	31	28	25	12	45	28	14	4
2013	21	27	0	58	24	51	18	5	11	48	23	13	4
2013	22	29	0	55	11	44	11	37	15	41	24	15	4
2013	23	25	0	0	12	58	22	5	12	41	23	14	4
2013	24	18	0	0	39	9	16	32	12	45	23	13	4
2013	25	22	0	16	69	24	8	22	9	51	21	13	4
2013	26	14	0	14	14	15	13	17	8	42	20	12	3
2013	27	19	0	0	0	10	46	11	8	41	16	12	4
2013	28	6	0	0	19	20	0	0	9	40	18	12	4
2013	29	5	0	16	21	0	0	8	7	39	16	11	3
2013	30	9	0	0	19	14	0	15	7	40	16	15	4
2013	31	11	0	0	0	7	24	15	6	39	14	13	3

2013	32	8	0	16	18	0	18	0	8	41	16	13	4
2013	33	3	0	0	18	7	0	0	10	46	18	15	5
2013	34	7	0	0	21	7	6	8	11	44	19	15	4
2013	35	16	0	0	39	13	35	0	10	39	19	14	3
2013	36	21	0	18	21	42	12	16	12	41	17	13	4
2013	37	12	0	19	19	7	11	15	12	37	19	12	5
2013	38	18	0	0	18	18	26	20	13	38	22	16	4
2013	39	35	62	0	47	49	38	18	20	43	32	19	6
2013	40	41	25	13	48	61	45	23	19	45	29	16	5
2013	41	32	88	0	47	52	23	15	21	41	28	17	5
2013	42	37	106	22	38	50	25	23	19	41	35	17	5
2013	43	43	116	46	34	46	42	22	16	39	36	15	4
2013	44	41	153	57	36	36	28	22	19	40	34	15	4
2013	45	54	175	94	40	52	41	23	21	41	33	17	4
2013	46	60	168	87	39	59	55	31	19	42	30	16	5
2013	47	74	235	74	59	84	62	40	20	40	33	19	5
2013	48	87	260	108	77	97	68	45	21	41	31	17	4
2013	49	95	303	112	66	109	82	44	25	44	34	18	5
2013	50	120	410	148	92	138	94	54	25	51	40	17	4
2013	51	133	367	159	110	167	107	66	28	50	37	21	7
2013	52	147	523	162	113	162	119	74	36	65	50	30	7
2014	1	202	458	211	183	211	198	131	42	68	53	33	9
2014	2	286	455	326	283	324	288	174	43	64	49	25	7
2014	3	350	867	548	293	368	308	157	38	57	45	22	7
2014	4	408	1244	729	337	379	323	162	39	64	45	26	6
2014	5	462	1324	872	394	421	385	168	39	62	45	26	7
2014	6	472	1299	942	398	441	378	162	37	60	45	25	6
2014	7	429	1197	850	321	413	335	180	37	61	43	25	6
2014	8	379	1103	657	284	381	290	159	38	63	44	25	6
2014	9	320	920	555	254	325	254	120	31	60	39	24	6
2014	10	274	738	482	201	278	218	123	30	59	40	23	6
2014	11	247	741	380	255	235	204	98	27	61	40	25	6
2014	12	192	590	344	141	182	140	95	28	62	41	23	6
2014	13	140	367	237	120	135	113	72	23	58	38	21	4
2014	14	122	326	222	108	123	92	54	20	54	38	19	5
2014	15	88	233	128	91	92	70	43	19	55	35	19	6
2014	16	69	200	101	53	73	50	44	16	50	33	17	5
2014	17	42	114	44	34	47	32	33	17	52	37	22	5
2014	18	23	0	0	25	33	17	26	16	52	32	19	4
2014	19	13	0	0	32	8	22	8	14	49	35	18	6
2014	20	25	0	31	0	35	25	26	14	51	35	18	4
2014	21	13	0	0	0	19	11	19	13	51	30	18	4
2014	22	12	0	48	0	30	4	0	12	48	29	14	4
2014	23	23	46	20	17	28	18	22	12	54	27	19	5
2014	24	17	0	17	33	29	14	6	12	50	26	15	4
2014	25	15	0	0	63	11	9	17	10	46	23	14	4
2014	26	8	0	0	19	21	0	7	10	43	21	14	4
2014	27	2	0	0	0	8	0	0	10	47	19	15	4

2014	28	23	0	20	40	27	21	20	9	48	19	14	4
2014	29	15	0	0	0	39	19	0	10	45	20	16	4
2014	30	11	0	0	0	15	12	15	7	42	20	14	4
2014	31	21	0	0	68	0	18	44	9	42	20	14	4
2014	32	16	0	0	0	14	22	25	10	53	20	17	5
2014	33	8	0	0	0	17	0	16	9	53	22	19	5
2014	34	19	0	0	79	0	44	0	11	53	21	19	4
2014	35	29	0	0	72	50	20	24	11	46	21	16	5
2014	36	17	0	0	37	37	10	11	12	45	22	15	4
2014	37	26	0	0	86	31	26	16	12	42	20	15	5
2014	38	23	0	0	74	13	22	27	16	40	25	17	5
2014	39	32	0	20	19	67	32	13	17	48	29	20	6
2014	40	27	0	54	0	29	33	23	18	48	34	22	5
2014	41	26	0	41	18	18	40	18	22	48	38	23	6
2014	42	30	66	45	17	24	29	28	22	49	39	22	6
2014	43	42	135	65	30	49	30	19	23	50	36	22	6
2014	44	47	136	57	46	45	46	28	22	45	37	21	6
2014	45	64	194	71	48	76	53	37	23	47	38	21	6
2014	46	62	171	71	55	70	61	26	25	48	43	20	5
2014	47	76	273	119	49	80	54	37	24	51	40	21	5
2014	48	90	286	121	68	106	73	40	39	52	43	21	6
2014	49	96	296	116	103	110	84	34	27	52	40	24	5
2014	50	115	362	186	93	124	89	50	27	53	46	23	6
2014	51	153	537	241	114	147	133	63	30	61	50	27	5
2014	52	147	458	233	94	164	117	74	38	79	68	37	7
2015	1	259	523	334	234	286	240	172	55	87	76	47	9
2015	2	443	706	591	394	538	444	237	59	85	73	43	8
2015	3	649	1601	1231	555	637	575	282	71	92	68	40	9
2015	4	818	2034	1907	663	763	666	304	98	100	74	46	8
2015	5	752	1752	1625	594	705	644	307	83	91	65	41	9
2015	6	704	1637	1416	582	686	630	303	80	91	65	41	7
2015	7	591	1344	1127	474	559	558	270	70	84	62	39	8
2015	8	490	1038	835	371	485	475	246	60	83	58	34	8

M099y: all-age ILI morbidity per 100,000 inhabitants; M04y: ILI morbidity per 100,000 inhabitants in the age-class of 0–4 years; M514y: ILI morbidity per 100,000 inhabitants in the age-class of 5–14 years; M1524y: ILI morbidity per 100,000 inhabitants in the age-class of 15–24 years; M2544y: ILI morbidity per 100,000 inhabitants in the age-class of 25–44 years; M4564y: ILI morbidity per 100,000 inhabitants in the age-class of 45–64 years; M6599y: ILI morbidity per 100,000 inhabitants in the age-class of ≥ 65 years; GTinf: Google trends relative query volume of *Influenza* search term; GTfev: Google trends relative query volume of *Fever* search term; GTcou: Google trends relative query volume of *Cough* search term; GTtac: Google trends relative query volume of *Tachipirina* search term; GTpar: Google trends relative query volume of *Paracetamol* search term.

Data Sources: Google Trends (www.google.com/trends), Inter-University Centre for Research on Influenza and other Transmissible Infections (CIRI-IT) (www.cirinet.it/jm/en/).

Box A. Example of R code used for the analysis

```
> trainset <- ILIdata[1:156,]
> sqrtM099y <- sqrt(trainset$M099y)
> require(AICcmodavg)
> Cand.models <- list( )
> Cand.models[[01]] <- lm(sqrtM099y ~ GTinf, data=trainset)
> Cand.models[[02]] <- lm(sqrtM099y ~ GTfev, data=trainset)
> Cand.models[[03]] <- lm(sqrtM099y ~ GTcou, data=trainset)
> Cand.models[[04]] <- lm(sqrtM099y ~ GTtac, data=trainset)
```

```
> Cand.models[[05]] <- lm(sqrtM099y ~ GTpar, data=trainset)
> Cand.models[[06]] <- lm(sqrtM099y ~ GTinf+GTfev, data=trainset)
> Cand.models[[07]] <- lm(sqrtM099y ~ GTinf+GTcou, data=trainset)
> Cand.models[[08]] <- lm(sqrtM099y ~ GTinf+GTtac, data=trainset)
> Cand.models[[09]] <- lm(sqrtM099y ~ GTinf+GTpar, data=trainset)
> Cand.models[[10]] <- lm(sqrtM099y ~ GTfev+GTcou, data=trainset)
> Cand.models[[11]] <- lm(sqrtM099y ~ GTfev+GTtac, data=trainset)
> Cand.models[[12]] <- lm(sqrtM099y ~ GTfev+GTpar, data=trainset)
> Cand.models[[13]] <- lm(sqrtM099y ~ GTcou+GTtac, data=trainset)
> Cand.models[[14]] <- lm(sqrtM099y ~ GTcou+GTpar, data=trainset)
> Cand.models[[15]] <- lm(sqrtM099y ~ GTtac+GTpar, data=trainset)
> Cand.models[[16]] <- lm(sqrtM099y ~ GTinf+GTfev+GTcou, data=trainset)
> Cand.models[[17]] <- lm(sqrtM099y ~ GTinf+GTfev+GTtac, data=trainset)
> Cand.models[[18]] <- lm(sqrtM099y ~ GTinf+GTfev+GTpar, data=trainset)
> Cand.models[[19]] <- lm(sqrtM099y ~ GTfev+GTcou+GTtac, data=trainset)
> Cand.models[[20]] <- lm(sqrtM099y ~ GTfev+GTcou+GTpar, data=trainset)
> Cand.models[[21]] <- lm(sqrtM099y ~ GTcou+GTtac+GTpar, data=trainset)
> Cand.models[[22]] <- lm(sqrtM099y ~ GTinf+GTfev+GTcou+GTtac, data=trainset)
> Cand.models[[23]] <- lm(sqrtM099y ~ GTfev+GTcou+GTtac+GTpar, data=trainset)
> Cand.models[[24]] <- lm(sqrtM099y ~ GTinf+GTfev+GTcou+GTtac+GTpar, data=trainset)
> Modelnames <- paste("model", 1:length(Cand.models), sep = "")
> aictab(cand.set = Cand.models, modnames = Modelnames, second.ord = TRUE, nobs = NULL, sort = TRUE)
> resM17 <- resid(Cand.models[[17]])
> acf(resM17)
> pacf(resM17)
> require(nlme)
> summary(GLS1 <- gls(sqrtM099y ~ GTinf+GTfev+GTtac, correlation = corARMA(p=1), method="ML", data=trainset))
> summary(GLS2 <- gls(sqrtM099y ~ GTinf+GTfev+GTtac, correlation = corARMA(p=1), method="REML", data=trainset))
> validset <- ILIdata[157:175,]
> predictM099yGLS <- predict(GLS1,validset)
> require(forecast)
> accuracy(predictM099yGLS,sqrt(validset$M099y))
> require(tseries)
> tsM099y <- ts(sqrtM099y, start=c(2011,42), frequency=52)
> HWM099y <- HoltWinters(tsM099y, seasonal="additive")
> predictM099yHW <- predict(HWM099y, n.ahead=19)
> accuracy(predictM099yHW, sqrt(validset$M099y))
```
